# Tryptophan Promotes the Production of Xanthophyll Compounds in Yellow Abdominal Fat through *HAAO*

**DOI:** 10.3390/ani14111555

**Published:** 2024-05-24

**Authors:** Xiaojing Liu, Lilin Men, Yanji Chen, Yongli Wang, Yanke Wang, Xu Zhang, Huanxian Cui, Yuming Guo, Jie Wen

**Affiliations:** 1 State Key Laboratory of Animal Biotech Breeding, State Key Laboratory of Animal Nutrition and Feeding, Institute of Animal Science, Chinese Academy of Agricultural Sciences (CAAS), Beijing 100193, China; 18810610203@163.com (X.L.); m18705430400@163.com (L.M.); bb369200773@163.com (Y.C.); 13592578118@163.com (Y.W.); yanke1222@163.com (Y.W.); zhangxu_caas@163.com (X.Z.); cuihuanxian@caas.cn (H.C.); 2 State Key Laboratory of Animal Nutrition, College of Animal Science and Technology, China Agricultural University, Beijing 100193, China

**Keywords:** chicken, abdominal fat color, xanthophyll compounds, tryptophan, *HAAO*

## Abstract

**Simple Summary:**

The abdominal fat of broiler chickens was used as one of the important parts for color evaluation, which directly affects the market price and demand of broilers. This study aimed to identify the contributors and the regulatory network involved in the formation of yellow and white color in abdominal fat. Four xanthophyll compounds were generally significantly correlated with eight metabolites. The high expression of 3-hydroxyanthranilate 3,4-dioxygenase (*HAAO*) in the yellow abdominal fat group leads to the degradation of tryptophan and its intermediate 5-hydroxyindole compounds, and subsequently to the formation of the four xanthophyll compounds. This study provides a genetic theoretical basis for improving the external appearance and internal quality of chicken abdominal fat.

**Abstract:**

Abdominal fat, which in the past was often regarded as waste and discarded, has in recent years been used as a fat source to produce meat by-products. Yellow abdominal fat has higher economic value. Therefore, improving the color of abdominal fat plays an important role in improving the appearance of meat products. This study aimed to identify the contributors and the regulatory network involved in the formation of yellow and white color in abdominal fat. We found that four xanthophyll compounds were significantly different in yellow and white abdominal fat chicken, including zeaxanthin, lutein, canthaxanthin, and β-cryptoxanthin. There were 551 different and 8 common metabolites significantly correlated with these 4 xanthophyll compounds. Similarly, a total of 54 common genes were identified in 4 common related pathways (Complement and coagulation cascades, Metabolic pathways, PPAR signaling pathway, Carbon metabolism) of the 8 common metabolites. The high expression of *HAAO* in the yellow abdominal fat group leads to the degradation of tryptophan and its intermediate 5-hydroxyindole, and subsequently to the formation of the four xanthophyll compounds. This process is also regulated by tyrosine, kynurenine 3-monooxygenase (KMO), homogentisate 1, 2-dioxygenase (HGD), etc. Together, these findings show the effect of tryptophan on abdominal fat color, as well as a negative regulatory effect of *HAAO* and 5-hydroxyindole on the production of xanthophyll compounds involved in abdominal fat coloration.

## 1. Introduction

The broiler industry is an important part of animal husbandry. Chicken, as an im-portant protein source in the human diet, has unique biological characteristics and irre-placeable economic value [1,2]. Influenced by traditional consumer culture, color has become one of the key indicators for consumers to evaluate the organoleptic quality of yellow-feathered broilers. The skin, tibiae, and abdominal fat of broiler chickens are usually used as important parts for the evaluation of their color, which directly affects the market price of and demand for broilers [3,4]. Consumers prefer broiler chickens with yellower skin, demanding yellow skin, yellow toes, and yellow fat. Therefore, for yellow-feathered broilers, which are mainly sold chilled, improving their color appearance is important for their market value.

Carotenoids are important fat-soluble pigments that affect the skin color of chicken carcasses, and β-carotene has the highest concentration of carotenoids found in poultry, mainly composed of lutein [5]. Lutein can inhibit the free radial-induced lipid peroxidation reaction and functions as an antioxidant. Some studies have reported that it may affect lipid metabolism and the oxidative stress response in chickens [6,7], and it cannot be de novo synthesized in broiler chickens. Therefore, natural lutein is often added to feed in the production of broilers to improve the color of their skin, tibia, abdominal fat, and other parts [8]. Many studies on coloration in broilers have also taken the effect of chicken abdominal fat color as an important index to evaluate pigment deposition in the body of broiler chickens [9]. In the past, abdominal fat was often regarded as waste with low economic value and discarded, but the large amount of abdominal fat waste generated would cause environmental problems and resource waste. Considering its high content of unsaturated fatty acids (UFAs), in recent years, abdominal fat has been increasingly used as a source of fat for the production of chicken sausage or other meat by-products [10]. Therefore, improving the color of abdominal fat can also play an important role in im-proving the appearance of meat products.

At present, most studies on improving the color of chicken abdominal fat involve adding exogenous feed. Many studies in the literature reported that exogenous addition of natural lutein, lobster meal, and other compounds can effectively improve the skin color of chicken carcass and the yellowness value of abdominal fat [11,12]. However, the high cost of supplemented feeds and the low deposition rate of lutein in feed ingredients highlight the importance of promoting the deposition of carotenoid compounds from the perspective of genetic regulation, thereby improving the color appearance of broilers. Gao et al. [13] analyzed the gene expression profile in the skin of golden pheasant by RNA sequencing (RNA-seq) and found that the expression of glutathione S-transferase alpha 2 (*GSTA2*) and apolipoprotein D (*APOD*) contributed to the deposition of carotenoids. Amengual et al. [14] showed that the color of the skin, abdominal fat, and other parts of broilers was regulated by beta-carotene oxygenase 1 (BCO1, also known as Breast color in chicken and as beta-carotene oxygenase 1 (BCO1) in human), which is an enzyme that can cleave and transfer carotenoids, which are then deposited in skin, fat, and other tissues to form yellow fat; it can also work together with melanin, which is synthesized by broilers themselves, to further regulate epidermal coloration [15,16]. Ren et al. [6] studied the effects of exogenous lutein on chicken skin yellowness, antioxidant properties, plasma metabolites, and liver gene expression by liver RNA-seq, as well as plasma metabolomics. However, their study focused on liver oxidative metabolism, and lacked systematic re-search on carotenoid transformation and deposition, which affect the coloration of the chicken. Elucidating the regulatory network of related metabolites is more conducive to the identification of the key genes and pathways involved in the regulation of the expression of carotenoids.

In order to elucidate the metabolic pathways of carotenoid deposition and to identify additional candidate genes involved in the regulation of color formation, we used Jinling yellow chickens as experimental materials and divided them into two groups (yellow and white abdominal fat) based on the yellowness of their abdominal fat. High perfor-mance liquid chromatography (HPLC) was performed to identify the differentially metab-olites in yellow and white abdominal fat. In addition, the differences in gene expression levels in abdominal fat of different yellowness were analyzed based on RNA-seq data analysis. The carotenoids and their related metabolites affecting the yellowness of chicken abdominal fat were identified by integrating metabolomics and transcriptomics analyses, and the candidate key genes with important pathways regulating the deposition of carot-enoids in abdominal fat were screened. This study provides a genetic theoretical basis for improving the external appearance and internal quality of chicken abdominal fat.

## 2. Materials and Methods

### 2.1. Animals and Sample Collection

Jinling colorful chickens bred by Guangxi Jinling Agricultural and Livestock Group Co., Ltd. (Nanning, China) were used as experimental material in this study. All chickens were raised in the same environment, with ad libitum feeding and drinking. The experimental diet was formulated by the company and its formulation and nutritional composition are shown in Table S1. After 56 days of feeding, chickens were slaughtered, and adipose tissue samples were collected from 10 randomly selected individuals with white adipose tissue (W-AF) and 10 randomly selected individuals with yellow adipose tissue (Y-AF). The collected tissue samples were frozen in liquid nitrogen and stored in a freezer at −80 °C until used for the determination of carotenoids, transcriptome analysis, and metabolome analysis. All experimental protocols were approved by the Science Research Department (which oversees animal welfare issues) of the Institute of Animal Sciences, Chinese Academy of Agricultural Sciences (Beijing, China) (No. IAS2019-21).

### 2.2. Determinationt of Carotenoids

The abdominal fat tissue samples stored at −80 °C were ground to powder using a ball mill (set at 30 Hz, 1 min). Then, 50 mg of each ground sample was extracted with 0.5 mL of a mixture of n-hexane/acetone/ethanol (1:1:1, *v*/*v*/*v*) containing 0.01% butylated hy-droxytoluene (BHT) (g/mL) [17,18]. Samples that had undergone preliminary processing were subjected to determination of carotenoid compounds by Ultra High Performance Liquid Chromatography-Tandem Mass Spectrometry (UHPLC-MS/MS), using an AB Sciex ExionLC™ AD UHPLC system coupled with an AB Sciex QTRAP^®^ 6500+ Mass Spectrometer (AB Sciex LLC., Framingham, MA, USA), at Wuhan Metware Biotechnology Co., Ltd. (Wuhan, China) [19,20]. After obtaining the mass spectrometry analysis data for the different samples, the chromatographic peaks of all target compounds were integrated, and quantitative analysis was performed using standard curves.

The Variable Importance in Projection (VIP) was obtained based on the orthogonal projections to latent structures-discriminant analysis (OPLS-DA) model. Metabolites with Fold Change ≥ 2 or ≤0.5, *p* < 0.05 and VIP > 1 were selected to screen for differential metabolites (DMs).

### 2.3. Full-Spectrum Metabolomics Measurement and Data Analysis

The abdominal fat tissue samples stored at −80 °C were ground in liquid nitrogen. After pre-treatment, 200 μL of the supernatant of the samples were analyzed by liquid chromatography-mass spectrometry (LC-MS). The sample extract was extracted by UHPLC-electrospray ionization (ESI)-MS/MS using the AB Sciex ExionLC™ AD UHPLC system coupled with the AB Sciex QTRAP^®^ 6500+ Mass Spectrometer (AB Sciex LLC.). The LIT and QQQ scans were recorded on a triple quadrupole linear ion trap mass spectrometer (QTRAP), QTRAP^®^ LC-MS/MS system, equipped with ESI Turbo ion spray interface, operated in positive and negative ion modes. All operations and measurements were performed at the Wuhan Metware Biotechnology Co., Ltd. [21].

The mass spectrometry data were processed using the Analyst software (version 1.6.3; Applied Biosystems/Sciex, Foster City, CA, USA). After analyzing the mass spectrometry data of metabolites from different samples, peak area integration was performed on all mass spectrometry peaks of the compounds, and correction was completed. DMs were analyzed using the DESeq2 package in the R software (version 4.2), and metabolites with Fold Change ≥ 2 or ≤0.5, *p* < 0.05 and VIP > 1 were selected as significantly different metabolites.

### 2.4. Pearson Correlation Analysis

Based on Pearson correlation calculation, the correlation coefficient of the differential carotenoids between the two groups and the DMs was used to measure the metabolites closely related to carotenoids. The closely related significant metabolites were determined by statistical significance testing (*p* < 0.05). This analysis was performed using the free online data analysis platform OmicShare tool (http://www.omicshare.com/tools) (accessed on 30 April 2024).

### 2.5. RNA Sequencing and Quality Control

Total RNA was extracted from the samples using the TRIzol reagent (Invitrogen, Carlsbad, CA, USA). The purity and integrity of the isolated RNA were determined using an Implen NanoPhotometer^®^ NP80 UV/VIS Spectrophotometer (Implen GmbH, Munich, Germany) and an Agilent bioanalyzer 2100 (Agilent Technologies Inc., Santa Clara, CA, USA), respectively. Non-strand specific RNA sequencing libraries were prepared and sequenced on the Illumina HiSeq 2500 platform (Illumina Inc., San Diego, CA, USA). The cDNA libraries were sequenced on the Illumina sequencing platform by Genedenovo Biotechnology Co., Ltd. (Guangzhou, China). Fastp file was used for quality control of raw reads from offline machines by filtering low-quality data to obtain clean reads. The HISAT2 software [22] was used to compare the reference genome of Gallus gallus-6.0, reconstruct the transcript using Stringtie, and calculate the expression levels of all genes in each sample using the RSEM software (version 1.3.3; http://deweylab.biostat.wisc.edu/rsem/) (accessed on 3 April 2024).

### 2.6. Weighted Gene Co-Expression Network Analysis

The relationship between genes and DMs was investigated by conducting Weighted Gene Co-Expression Network Analysis (WGCNA) on the transcriptome data and differential genes using the “WGCNA” package in the R 4.0.5 software [23]. The package was used to load the transcriptome data, calculate the correlation between genes, construct an undirected weighted network based on the correlation matrix, and set the soft-thresholding power parameter (β) to 4 to ensure scale-free network properties and establish a weighted gene co-expression network. The WGCNA algorithm was used to detect modules within the gene co-expression network, and genes were clustered into different modules using the dynamic tree-cutting method. The differential metabolite data were integrated into the gene co-expression network to establish associations between genes and DMs. Pearson correlation analysis and/or other statistical methods were used to determine significant associations between modules and DMs. Gene significance (GS) was used to measure the relationship between genes and DMs, while Module Membership (MM) was used to represent the correlation of each gene with its respective module. Genes with |GS| = 0.25 and |MM| = 0.7 were further analyzed to identify hub genes within the modules.

### 2.7. Kyoto Encyclopedia of Genes and Genomes Pathway Enrichment Analyses

OmicShare tools (www.omicshare.com/tools) (accessed on 5 April 2024) were used for Kyoto Encyclopedia of Genes and Genomes (KEGG) pathway enrichment analysis of differentially expressed genes (DEGs), *Q* value < 0.05 in KEGG terms was considered as a significant signaling pathway.

### 2.8. Statistical Analysis

The data obtained in this study are expressed as the mean ± standard deviation (SD). Analysis of variance (ANOVA) and Tukey’s test were performed for multiple comparisons of means at a significance level of *p* < 0.05 using the GraphPad Prism 9.5 software (GraphPad Software Inc., San Diego, CA, USA).

## 3. Results

### 3.1. Determination of Phenotypic Traits in Yellow and White Abdominal Fat Groups

**Figure 1:**
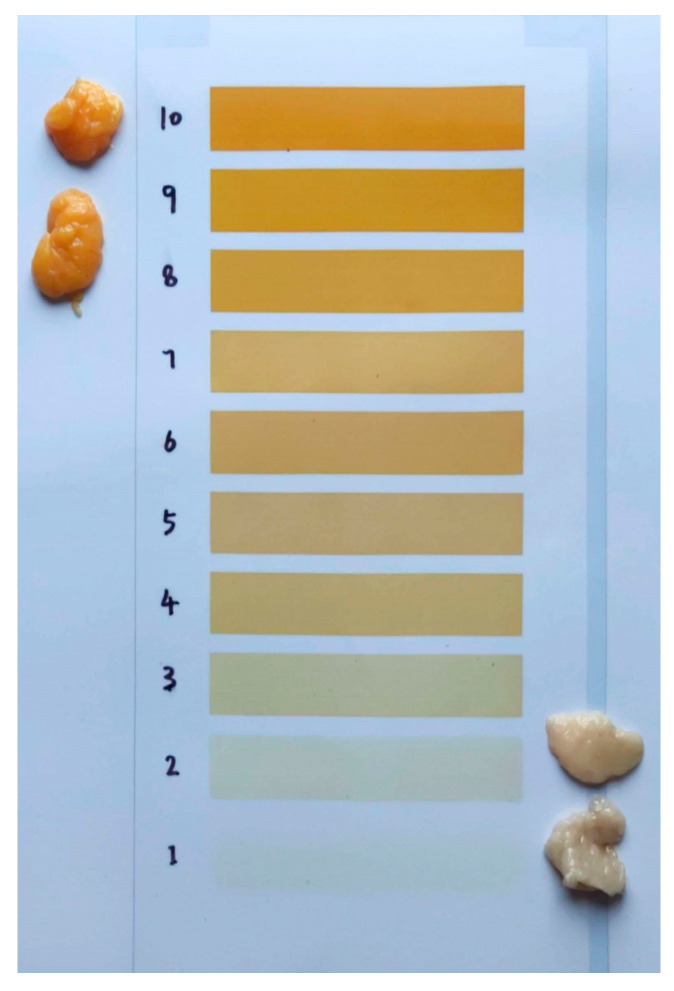
Plot of the color score of the groups. The color score of the yellow abdominal fat is 9 and 10, and that of the white abdominal fat is 1 and 2. Y-AF, yellow adipose tissue; W-AF, white adipose tissue.

As shown in Figure 1, the color contrast between yellow abdominal fat and white abdominal fat is very significant. The color score of the yellow abdominal fat is 9 and 10, while that of the white abdominal fat tends to be 1 and 2.

**Figure 2:**
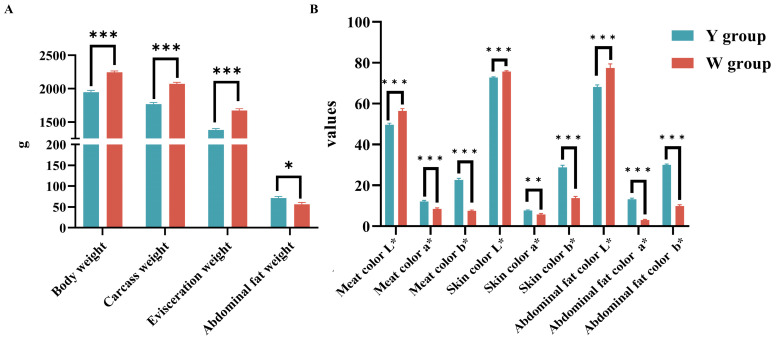
(**A**) The slaughter indexes weight (/g). (**B**) Three tissues (breast muscle, back skin, abdominal fat) color value (L* (brightness), a* (redness) and b* (yellowness)) of the Y-AF and W-AF groups. Y-AF, yellow adipose tissue; W-AF, white adipose tissue. *, *p* < 0.05; **, *p* < 0.01; ***, *p* < 0.001.

The slaughter index and the skin color of abdominal fat, breast muscle, and back skin were significantly different in the Y-AF and W-AF groups (Figure 2). Compared with the Y-AF group, body weight, carcass weight, evisceration weight, and breast muscle weight in the W-AF group were significantly increased (*p* < 0.01), while abdominal fat weight was significantly decreased (*p* < 0.01). The brightness values of the three tissues (breast muscle, back skin, and abdominal fat) were significantly increased (*p* < 0.01), and the redness and yellowness values were significantly decreased (*p* < 0.01).

### 3.2. Contribution of Carotenoids to the Abdominal Fat Color

A total of 18 carotenoids contributing to abdominal fat color were identified in the Y-AF and W-AF groups (Table 1).

**Figure 3:**
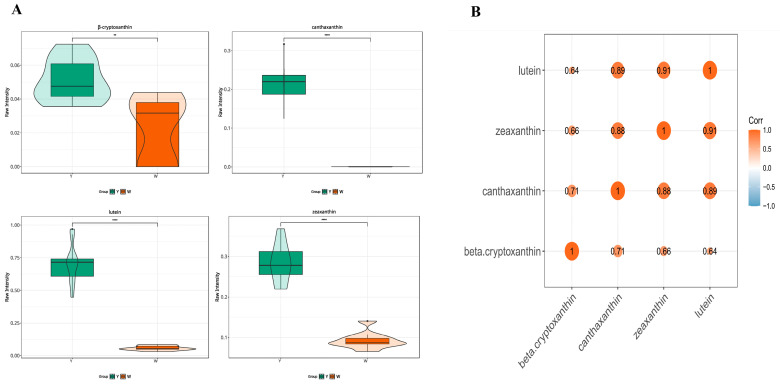
The content of four xanthophylls significant difference in the Y-AF and W-AF groups and their correlation. (**A**) The relative content of four xanthophylls in abdominal fat (μg/g). (**B**) The correlation between the relative content of four xanthophylls in abdominal fat (μg/g) was analyzed by Pearson correlation coefficient in the Y-AF and W-AF groups. Y-AF, yellow adipose tissue; W-AF, white adipose tissue. **, *p* < 0.01; ****, *p* < 0.0001.

Among the 18 identified carotenoids significantly contributing to abdominal fat color, 4 compounds were significantly different in the Y-AF and W-AF groups, namely zeaxanthin, lutein, canthaxanthin, and β-cryptoxanthin. The contents of the four compounds in the Y-AF group are significantly higher than those in the W-AF group, and all belong to the xanthophylls (Figure 3A). A phenotypic correlation analysis revealed the relationship between four substances (Figure 3B), indicating that the four compounds had a high positive correlation coefficient (all *p* < 0.05).

### 3.3. Regulate Key Metabolites to the Four Xanthophyll Compounds

**Figure 4:**
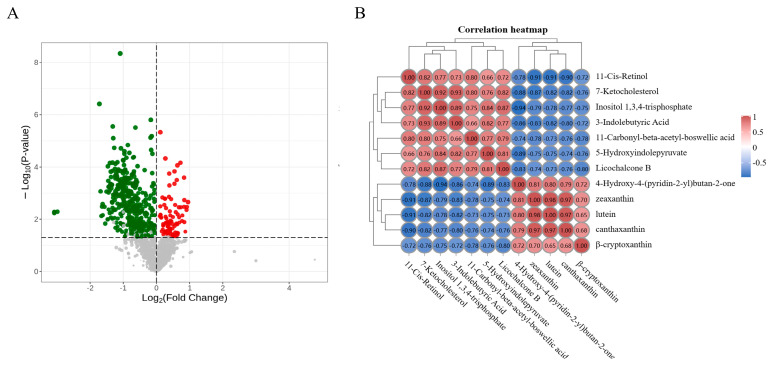
(**A**) Volcano plot of Y-AF and W-AF groups with metabolic data. Red represents up—regulated genes and green represents down—regulated genes. (**B**) The correlation between the relative content of four xanthophylls and eight common metabolites in abdominal fat was analyzed by Pearson correlation coefficient in the Y-AF and W-AF groups. Y-AF, yellow adipose tissue; W-AF, white adipose tissue. Red represents positive correlation, blue represents negative correlation.

We also examined the relationship between the four compounds contributing to the differences in the Y-AF and W-AF groups through abdominal fat metabolites. The metabolites were detected by LC-MS/MS, and a total of 1529 common metabolites were annotated (Table S2). We identified metabolites that regulate the Y-AF and W-AF groups by differential analysis. Cross-validation showed that the test model was not overfitted (R2 = 0.96, Q2 = −1.32). We extracted VIP values from the variable importance plot of the OPLS-DA model to identify differentially accumulated metabolites in the Y-AF and W-AF groups. DMs were identified according to these criteria. The DMs are shown in the volcano plot; a total of 551 DMs were obtained, of which 94 were upregulated and 457 were downregulated in the Y-AF group (Figure 4A, Table S3).

**Figure 5:**
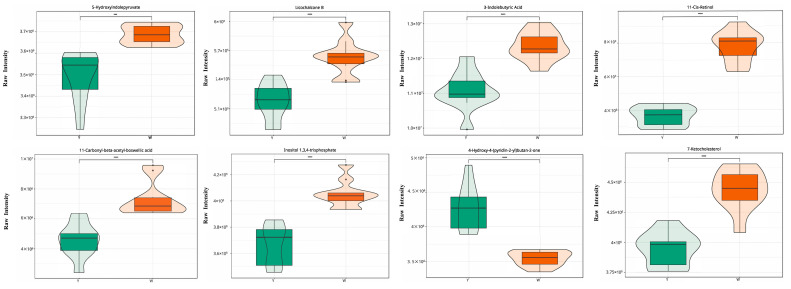
The content of eight common metabolites significant difference in the Y-AF and W-AF groups. Y-AF, yellow adipose tissue; W-AF, white adipose tissue. ***, *p* < 0.001; ****, *p* < 0.0001.

To identify metabolites that cause changes in the 4 key compounds, we performed correlation analysis of these 551 DMs and 4 compounds (Table S3). Among the 551 DMs, 8 (3-indolebutyric acid, 4-hydroxy-4-(pyridin-2-yl)butan-2-one, 5-hydroxyindolepyruvate, 7-ketocholesterol, 11-carbonyl-beta-acetyl-boswellic acid, 11-cis-retinol, inositol 1,3,4-trisphosphate, and licochalcone B) were commonly significantly correlated with the 4 compounds (Figure 4B), so they were considered as the universal contributors to the color of abdominal fat. Among these eight compounds, seven were downregulated in the Y-AF group, except for 4-hydroxy-4-(pyridin-2-yl) butan-2-one, which was upregulated in the Y-AF group (Figure 5).

### 3.4. Genes and Pathways Associated with Eight Key Metabolites

**Figure 6:**
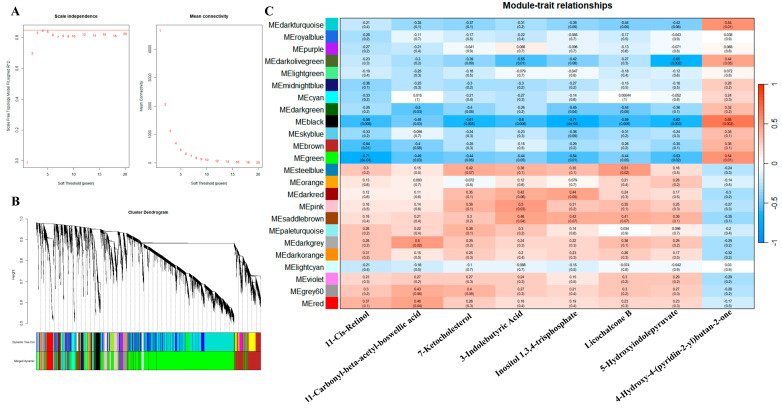
Co-expression network analysis of genes associated with eight metabolites in the Y-AF and W-AF groups. (**A**) Scale independence and mean connectivity. (**B**) The cluster of genes. (**C**) Module heatmap, the gene modules and trait relationships (red indicates positive correlation; blue indicates negative correlation). Y-AF, yellow adipose tissue; W-AF, white adipose tissue.

Four types of lutein compounds affecting yellow and white abdominal fat were identified in the previous stage, and eight key metabolites that can regulate the content of these four types of compounds were found. In order to better understand the gene and metabolite regulatory network affecting the color of abdominal fat, RNA-seq was performed on the two groups of individuals from the Y-AF and W-AF groups, and WGCNA was performed on the eight key metabolites and transcriptome data to obtain related genes and regulatory pathways (Figure 6A,B).

The WGCNA between 16,635 genes and the 8 identified important metabolites revealed that these 8 metabolites were mainly divided into two categories according to the significantly enriched modules, indicating the consistency with the phenotypic correlation analysis (Figure 6C). Genes associated with seven metabolites were enriched in the black module with significant negative correlation, and 4-hydroxy-4-(pyridin-2-yl) butan-2-one with the significant positive correlation. Similarly, genes associated with the 11-cis-retinol, 11-carbonyl-beta-acetyl-boswellic acid, inositol 1,3,4-trisphosphate and 5-hydroxyindolepyruvate were enriched in the green module with significant negative correlation, and 4-hydroxy-4-(pyridin-2-yl) butan-2-one with significant positive correlation. In addition, the details of the eight compounds that also have multiple significant positive or negative correlation modules are shown in Table 2.

### 3.5. Genes Markers and Pathways Related to the Important Contributors to the Color of Abdominal Fat

We investigated the gene markers and pathways related to the important contributors to the color of abdominal fat. Based on the above significantly enriched modules of different contributors by WGCNA, the significant genes associated with the eight key metabolites and their involvement in the pathways were screened (Table S4). Considering the eight metabolites as a whole, we found four common pathways. Subsequently, we returned to the four common pathways (Complement and coagulation cascades, Metabolic pathways, PPAR signaling pathway, Carbon metabolism) from eight metabolites, and the corresponding genes containing the main pathways were respectively screened (Table S5). The results identified a total of 54 common genes in 4 common related pathways of the 8 metabolites.

**Figure 7:**
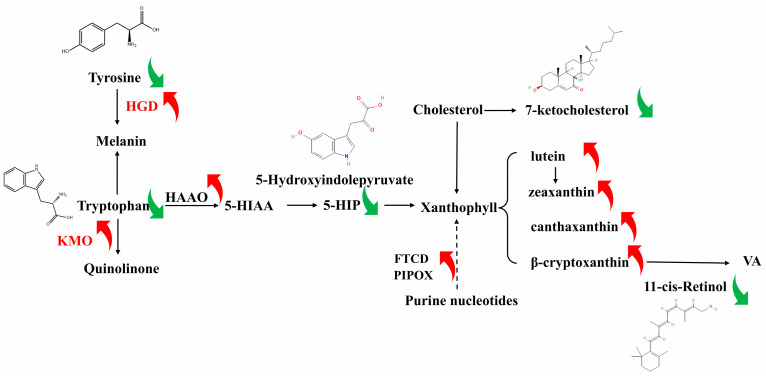
Network of xanthophylls production. Red and green arrows indicate upregulation and downregulation, respectively.

We also screened for hub genes among the 54 genes using the STRING and Cytoscape tools. After reviewing the relevant literature to identify gene markers that regulate abdominal fat color, we selected the top 20 genes for further investigation. The relevant regulatory network was constructed with xanthophyll compounds, key metabolites, and hub genes, as shown in Figure 7.

## 4. Discussion

The processing and use of chicken abdominal fat can increase the added value of poultry farming and reduce resource wastage, thus holding significance for agricultural economy and promoting industrial development. Consumers are more likely to choose yellow abdominal fat; thus, improving the appearance and color of abdominal fat is beneficial for consumer choice. However, which compounds play an important role in the formation of the yellow abdominal fat and how these compounds are metabolized have not been fully resolved. This study used LC-MS, transcriptomics, and metabolomics to simultaneously perform comparative analyses of yellow and white abdominal fat, focusing on the formation of abdominal fat color. We have identified four major contributors (lutein, zeaxanthin, canthaxanthin, and β-cryptoxanthin) to the color of abdominal fat, analyzed their relationship, and found their metabolic and gene markers and associated biochemical pathways.

Xanthophyll carotenoids play a unique role in poultry; they are responsible for the color of the skin of laying hens and egg yolk, which is the main reason to enrich feeds with carotenoids. Intensely colored chicken skin and egg yolks are perceived as healthier products by consumers in several countries, such as Mexico, China, and Bangladesh. This perception serves as an economic driver in the poultry industry [3,5]. Four xanthophyll compounds (lutein, zeaxanthin, canthaxanthin, and β-cryptoxanthin) were identified as important contributors to the color of abdominal fat by meeting two criteria of *p* < 0.05 and FC > 2. A significant positive relationship was found between the total content of carotenoids (lutein + zeaxanthin) and the yellow color of the breast meat [24], which is consistent with the results of this study. Compared with the W-AF group, the content of lutein + zeaxanthin in the Y-AF group is significantly increased. The xanthophyll carotenoids lutein and zeaxanthin accumulate in the yolks of laying hens fed a typical yellow maize-based and marigold feed; thus, they are used to improve color of eggs from laying hens [25]. Canthaxanthin is also widely used as a feed additive to improve skin color in poultry. The chicks that hatched from eggs laid by breeder hens fed the canthaxanthin supplementation diet showed a higher pigmentation colorimetric score of RYCF for their shank skin (*p* < 0.05) [26]. The provitamin A xanthophyll, β-cryptoxanthin, is a bipolar, oxygenated molecule that is highly bioavailable from supplements and food [27,28]. β-cryptoxanthin has apparent higher bioavailability than β-carotene in humans consuming mixed foods. Furthermore, yolks from hens fed maize selectively bred for increased β-cryptoxanthin content accumulate intact β-cryptoxanthin [29,30], which has provitamin A activity [27,31]. Therefore, we inferred that these four compounds had an important effect on color formation in abdominal fat.

We also examined the relationship between lutein, zeaxanthin, canthaxanthin, and β-cryptoxanthin from the perspective of the compounds and their metabolites. The emergence of metabolomics has promoted research breakthroughs in the biochemical formation mechanism of biological traits [32,33]. High throughput metabolomics technology is an effective means to screen the key metabolites of abdominal fat color [34], but systematic investigations are lacking on this project. Based on the correlation between the identified four compounds and DMs, the identification of common DMs. Through the above methods, we identified eight metabolites (3-indolebutyric acid, 4-hydroxy-4-(pyridin-2-yl)butan-2-one, 5-hydroxyindolepyruvate, 7-ketocholesterol, 11-carbonyl-beta-acetyl-boswellic acid, 11-cis-retinol, inositol 1,3,4-trisphosphate, and licochalcone B) that are co-related to lutein, zeaxanthin, canthaxanthin, and β-cryptoxanthin. In addition, gene regulation of key metabolites is also one of the important goals of abdominal fat research, which can be better applied to chicken production practice through gene selection and regulation. We thus performed WGCNA of key metabolites and transcriptome data to construct regulatory networks of metabolites and genes.

We propose a regulatory network in Figure 7. As a core amino acid, tryptophan, under the action of *HAAO*, can generate 5-HIAA and 5-hydroxyindolepyruvate. Among the eight key metabolites identified, the common source of 7-ketocholesterol and lutein is the cholesterol metabolic pathway, as cholesterol is one of the precursors of lutein, and the oxidation of cholesterol can produce 7-ketocholesterol [35]. Not only that, but tryptophan is also one of the precursors of lutein synthesis. Also, 3-indolebutyric acid is usually derived from tryptophan synthesis, while 5-hydroxyindolepyruvate is a key intermediate product in the tryptophan metabolism pathway [36], which may be involved in regulating pigment synthesis and metabolism process in organisms, and ultimately affect the generation and decomposition of pigments. The 3-hydroxyanthranilate 3,4-dioxygenase (*HAAO*) gene encodes a key enzyme in tryptophan metabolism that catalyzes the conversion of tryptophan to pyridinone acid. Tryptophan is also regulated by the enzyme encoded by the *KMO* gene, which catalyzes the conversion of tryptophan into the unique metabolite quinolinic acid in the tryptophan metabolic pathway [37]. An important role of the metabolic pathway of alanine is to participate in the process of tryptophan metabolism and can produce tryptophan. The beta-ureidopropionase 1 (*UPB1*) gene encodes an enzyme involved in the breakdown of urea pyruvate, converting it into 3-hydroxypyruvate and urea [38]. Urea pyruvate is one of the metabolites of amino acid alanine. Through the differential expression of *HAAO*, *KMO*, and *UPB1*, the genes coding enzyme may change, which leads to the change of metabolites content.

The *FTCD* gene encodes formimidoyltransferase cyclodeaminase, which can catalyze the conversion of methionine to N-formylmethionine and is then involved in purine metabolic pathways [39]. The synthesis of lutein involves intermediate products of purine metabolism. Briefly, purine nucleotides produce intermediate products such as adenylate and guanylate during purine metabolism. Guanylic acid can be converted to xylulose-5-phosphate through a series of enzymes, which then undergo a series of reactions ultimately leading to the conversion of lutein. The enzyme encoded by the pipecolic acid and sarcosine oxidase (*PIPOX*) gene is involved in the metabolism of uracil and dihydropyrimidine, which are intermediate products of purine metabolism. Gene mutation in *PIPOX* may lead to abnormal purine metabolism, and then cause related diseases [40].

Important metabolites and genes are not only closely related to the synthesis of amino acids, such as tryptophan and tyrosine, but also fatty acids. Inositol 1,3, 4-trisphosphate is a secondary signaling molecule that signals when cells are stimulated, specific cell membrane receptors activate phospholipase C (PLC) [41], that hydrolyzes phosphatidylinositol diphosphate (PIP2) to IP3 and diacylglycerol (DAG). IP3 binds to the IP3 receptor on the endoplasmic reticulum, inducing the release of calcium ions in the endoplasmic reticulum, resulting in an increase in the intracellular calcium concentration. The *PLA2G12B* gene encodes a member of the phospholipase A2 family, which catalyzes the hydrolysis of phospholipids, such as phosphatidylcholine on cell membranes, to produce free fatty acids and dissolved phospholipids [42]. *FABP1* gene encodes the fatty acid-binding protein 1 (FABP1), which is highly expressed in the liver and can bind and transport fatty acids. It is involved in the uptake, transport, and metabolism of fatty acids in cells, and promotes the utilization and oxidation of fatty acids [43].

In addition, we detected several genes DEGs related to coagulation function (*SERPINC1*, *SERPINF2*, *SERPIND1*, *FGA*, *FGG*, *FGB*, etc.) and inflammation (*MASP2*, *CFI*, *C4*), but there are no detailed reports confirming that these processes are related to lutein compounds. However, in this study, it is associated as a hub gene, indicating that it has an important role in these processes, and further studies are needed to explore its specific function.

In summary, based on the difference and correlation analysis of the omics data, the regulatory network of genes and metabolites was constructed, but the corresponding molecular biological verification needs to be performed in the future.

## 5. Conclusions

Overall, we found a relationship between four xanthophyll compounds and amino acids, especially tryptophan. 5-Hydroxyindole is a key compound in the synthesis of tryptophan: it is differentially expressed and significantly associated with four xanthophyll substances (zeaxanthin, lutein, canthaxanthin, β-cryptoxanthin). In addition, the intramolecular dioxygenase encoded by the *HAAO* gene regulated the content of the tryptophan intermediate 5-hydroxyindole, resulting in an increase of the content of four lutein compounds and the formation of yellow abdominal fat. This process is also regulated by tyrosine, KMO, HGD, and other proteins. Our study indicates the regulatory effect of tryptophan on the production of xanthophyll compounds, further revealing the regulatory mechanism of yellow abdominal fat.

## Figures and Tables

**Table 1:** Content of 18 xanthophylls compounds determined by Y-AF and W-AF groups.

Compounds (ug/g)	Class	Y-AF Group	W-AF Group	*p*-Value
valueviolaxanthin myristate	xanthophylls	2.332 ± 0.157	2.484 ± 0.173	0.05
zeaxanthin palmitate	xanthophylls	3.295 ± 0.362	3.317 ± 0.751	0.93
zeaxanthin	xanthophylls	0.284 ± 0.048 ^a^	0.093 ± 0.021 ^b^	9.3 × 10^−10^
lutein	xanthophylls	0.71 ± 0.157 ^a^	0.058 ± 0.017 ^b^	1.3 × 10^−10^
canthaxanthin	xanthophylls	0.215 ± 0.053 ^a^	0.001 ± 0 ^b^	1.7 × 10^−10^
lutein caprate	xanthophylls	0.026 ± 0.009	0.026 ± 0.006	0.95
lutein stearate	xanthophylls	0.309 ± 0.08 ^a^	0.452 ± 0.065 ^b^	0.0004
5,6epoxy-lutein-caprate-palmitate	xanthophylls	14.506 ± 3.166	15.608 ± 3.966	0.5
lutein dipalmitate	xanthophylls	3.458 ± 1.031	3.101 ± 0.71	0.38
lutein oleate	xanthophylls	0.349 ± 0.059	0.393 ± 0.072	0.14
rubixanthin palmitate	xanthophylls	2.791 ± 0.766	2.691 ± 0.525	0.74
violaxanthin dipalmitate	xanthophylls	0.047 ± 0.016	0.055 ± 0.013	0.21
β-cryptoxanthin palmitate	xanthophylls	3.44 ± 0.967	2.835 ± 0.368	0.08
violaxanthin-myristate-caprate	xanthophylls	0.079 ± 0.034	0.096 ± 0.04	0.32
violaxanthin	xanthophylls	0.006 ± 0.001	0.003 ± 0.003	0.05
lutein distearate	xanthophylls	0.181 ± 0.129	0.125 ± 0.073	0.25
neochrome palmitate	xanthophylls	0.304 ± 0.215	0.24 ± 0.213	0.52
β-cryptoxanthin	xanthophylls	0.051 ± 0.013 ^a^	0.022 ± 0.019 ^b^	0.001
SUM	xanthophylls	1.26 ± 0.243 ^a^	0.173 ± 0.041	4.5 × 10^−11^

SUM: The sums of the four xanthophylls (zeaxanthin + lutein + canthaxanthin + β-cryptoxanthin). Y-AF, yellow adipose tissue; W-AF, white adipose tissue. a and b indicated statistically significant differences between the two groups (*p* < 0.05).

**Table 2:** Modules significantly related to eight metabolites.

ID	11-cis-ROL	AKBA	7-KC	IBA	IP3	LCB	5-HIP	HPB
MEblack	−0.58	−0.48	−0.61	−0.6	−0.71	−0.59	−0.62	0.65
MEgreen	−0.71	−0.49			−0.54		−0.53	0.54
MEbrown	−0.54							
MEdarkgreen		−0.5			−0.45			
MEdarkgrey		0.5						
MEred		0.46						
MEsaddlebrown				0.46				
Mepink				0.5				
MEdarklivegreen				−0.55			−0.65	
Mesteelblue						0.51		
Medarkturquoise								0.54

11-cis-ROL: 11-Cis-Retinol; AKBA: 11-Carbonyl-beta-acetyl-boswellic acid; 7-KC: 7-Ketocholesterol; IBA: 3-Indolebutyric Acid; IP3: Inositol 1,3,4-trisphosphate; LCB: Licochalcone B; 5-HIP: 5-Hydroxyindolepyruvate; HPB: 4-Hydroxy-4-(pyridin-2-yl) butan-2-one.

## Data Availability

The raw sequence data reported in this paper have been deposited in the Genome Sequence Archive in National Genomics Data Center, China National Center for Bioinformation/Beijing Institute of Genomics, Chinese Academy of Sciences (GSA-Chicken: CRA016381) that are publicly accessible at https://ngdc.cncb.ac.cn/gsa-chicken (accessed on 3 May 2024).

## Supplementary Materials

The following supporting information can be downloaded at: https://www.mdpi.com/article/10.3390/ani14111555/s1, Table S1: Composition and main characteristics of feed; Table S2: The metabolites were detected of Y-AF and W-AF group; Table S3: The screened DMs between Y-AF and W-AF group; Table S4: The screened pathways about 8 key metabolites; Table S5: The genes associated with four common pathways of 8 DMs enrichment.
